# Study protocol of the e-Intervention Enhancing Mental Health in Adolescents (IMPROVA) project: a randomised controlled trial to promote adolescents’ mental health and well-being in four European countries

**DOI:** 10.1136/bmjopen-2025-108674

**Published:** 2026-04-28

**Authors:** Rodrigo Antunes Lima, Harald Baumeister, Rüdiger Pryss, Ann-Marie Küchler, Robin Kraft, Nils Berghoff, Luzia Nagel, Manfred Reichert, Abdul Rahman Idrees, Michael Stach, Maria Melchior, Camille Davisse-Paturet, Emma Falkiner, Judith van der Waerden, Adriana Baban, Diana Taut, Ingrid Danila, Mariona Perez-Anton, Daniele Porricelli, Julia Sanguesa, Maria Victoria Moneta, Rocío García-Carrión, Catrin Finkenauer, Gonneke WJM Stevens, Lydia Krabbendam, Felix Bolinski, Petra Maresova, Per Nilsen, Nadine Karlsson, Natalia Moreno, Anaïs Le Corvec, Josep Maria Haro

**Affiliations:** 1 Impact and Prevention of Mental Disorders Research Group, Institut de Recerca Sant Joan de Déu (IRSJD), Esplugues de Llobregat, Spain; 2 Centro de Investigación Biomédica en Red de Salud Mental (CIBERSAM), Instituto de Salud Carlos III, Madrid, Spain; 3 Clinical Psychology and Psychotherapy, University of Ulm, Ulm, Germany; 4 Partner site Mannheim-Heidelberg-Ulm, German Center for Mental Health (DZPG), Ulm, Germany; 5 Partner site Ulm, German Center for Child and Adolescent Health (DZKJ), Ulm, Germany; 6 Institute of Medical Data Science, University Hospital Wurzburg, Würzburg, Germany; 7 Institute of Clinical Epidemiology and Biometry, University of Würzburg, Würzburg, Germany; 8 Institute of Databases and Information Systems, Ulm University, Ulm, BW, Germany; 9 INSERM, Pierre Louis Institute of Epidemiology and Public Health, IPLESP, Social Epidemiology, Mental Health and Addictions, ESSMA, Sorbonne Université, Paris, France; 10 Pierre Louis Institute of Epidemiology and Public Health, IPLESP, Social Epidemiology, Mental Health and Addictions, ESSMA, Sorbonne Université, INSERM, Paris, France; 11 Department of Psychology, Babes-Bolyai University, Romania, Cluj-Napoca, Romania; 12 Departament de Psicologia Clínica i de la Salut, Universitat Autonoma de Barcelona, Bellaterra, Spain; 13 Department of Education, University of Deusto, Bilbao, Spain; 14 Ikerbasque, Basque Foundation for Science, Bilbao, Spain; 15 Department of Interdisciplinary Social Science, Utrecht University, Utrecht, Netherlands; 16 Department of Clinical, Neuro-and Developmental Psychology, Vrije Universiteit Amsterdam, Amsterdam, Netherlands; 17 Department of Public Mental Health, Trimbos Instituut, Netherlands Institute of Mental Health and Addiction, Utrecht, Netherlands; 18 Betthera s.r.o, Hradec Králové, Czech Republic; 19 Department of Health, Medicine and Caring Sciences, Linköping University, Linköping, Sweden; 20 Ajuntament de Sant Boi de Llobregat, Sant Boi de Llobregat, Spain; 21 Cliclab Transformative Agent SL, Barcelona, Spain

**Keywords:** Adolescents, Psychosocial Intervention, Community child health, eHealth, MENTAL HEALTH

## Abstract

**Introduction:**

This trial aims to evaluate the impact of the IMPROVA intervention programme in improving mental health, quality of life, and well-being in adolescents enrolled in secondary schools in four European countries.

**Methods and analysis:**

The IMPROVA intervention will be evaluated using a two-arm parallel group pragmatic cluster randomised controlled trial with an intervention and a wait-list control group. Secondary schools in France, Germany, Romania, and Spain were recruited. Originally, we estimated to enrol a total of 6000 students within 64 schools; that is, 16 schools per country. The IMPROVA programme is a multi-level intervention that provides tailored content for adolescents, families, and school staff. This content creates a unified and supportive framework that promotes mental health and social-emotional development among adolescents. A series of implementation strategies was planned to support the uptake of the programme into the education setting and among participants. Study outcomes were assessed at baseline, mid-term (during the intervention), postintervention (primary end point), and will be assessed at postintervention (secondary end-point; 6 months after postintervention). Overall mental health (Strengths and Difficulties Questionnaire) is the primary outcome. Secondary outcomes include: health-related quality of life, depression, anxiety, social isolation, and self-esteem. The trial will be evaluated regarding its effectiveness, cost-effectiveness, implementation, and social return on investment analysis.

**Ethics and dissemination:**

This study has received the approval of human research ethics committees in France (Comité de Protection des Personnes Ile-de-France VIII”, No. 2024-A00201-46), Germany (Ulm University Ethics Committee, No. 186-24), Romania (The Research Ethics Subcommittee of the Babeș-Bolyai University of Cluj-Napoca, No. 14.146/23.09.2024), and Spain (CEIm Fundació Sant Joan de Déu, No. PIC-61-24). Results will be disseminated through peer-reviewed open-access publications and presentations at national and international conferences. Non-technical summaries will be shared with public health authorities, participants, and stakeholder organisations. All findings will be reported in aggregate form, ensuring no individual participant can be identified.

**Trial registration number:**

NCT06556576.

**STRENGTHS AND LIMITATIONS OF THIS STUDY**

- A key strength of this study is its rigorous cluster randomised controlled trial design and the theory-driven intervention design grounded in social and emotional learning and cognitive-behavioural frameworks.
- The IMPROVA programme is developed using a co-creation approach that systematically integrates input from students, school staff and families through qualitative and pilot-testing activities across France, Germany, Romania and Spain.
- The study has sufficient statistical power to detect changes in primary and secondary outcomes and to support planned effect-modification analyses.
- Alongside analyses of effectiveness and effect pathways, the study incorporates a comprehensive evaluation of implementation processes, economic impact and social return on investment.
- A potential major limitation of the IMPROVA programme is the inherent complexity of achieving its systematic integration into school curricula and daily routines across four European countries, a challenge for which multiple implementation strategies have been proactively developed.

## Introduction

 Promoting adolescents’ mental health is pivotal. Notably, approximately half of all mental health problems in adulthood have their onset before or during adolescence.^1^ Furthermore, young people who experience mental health problems before the age of 14 years are more likely to report mental burdens in adulthood.^2^ In addition, mental health problems during adolescence are associated with a higher risk of suicide, which is the second most common cause of death among individuals aged 15–29 years worldwide.^3^ Finally, depression and anxiety are among the top five causes of overall disease burden in this age group.^4^

Schools represent a fundamental setting for implementing interventions to promote mental health and prevent psychological problems in adolescence.^5^ However, most systematic reviews indicate that primary interventions implemented in school settings have weak to modest effects.^6^,^8^ Among the various approaches, universal social and emotional learning (SEL) interventions have the strongest evidence of enhancing young people’s social and emotional skills while also reducing symptoms of depression and anxiety in the short term.^8^

Furthermore, cognitive-behavioural therapy (CBT)-based interventions, both universal and targeted, have demonstrated effectiveness in reducing internalising and externalising symptoms.^69^,^12^ While preventive universal CBT programmes are promising in alleviating symptoms of depression and anxiety in the short term, targeted CBT interventions seem particularly appropriate for young people experiencing mild but detectable depressive symptoms, with effects extending into the medium term (ie, 6 months after the intervention period).^10^ Additionally, while teachers can effectively deliver universal interventions,^13^ there is no strong evidence that teacher-led programmes adequately address the needs of students experiencing symptoms of depression or anxiety.^13^ For these students, CBT-based interventions delivered by external professionals, such as psychologists, provide the most convincing evidence of improving mental health outcomes.^14^ However, the high costs associated with incorporating psychologists into community-based interventions, such as in schools, pose a significant barrier to large-scale implementation.^15^ This challenge underscores the importance of specialised training of teachers or other professionals (eg, primary healthcare, other school staff) in addressing both emerging and more severe mental health difficulties. Considering the state-of-the-art on mental health promotion among youth,^6^,^12^ intervention programmes should integrate SEL’s broad skill-building benefits with CBT’s symptom-focused strategies to create a comprehensive and effective school-based mental health intervention that addresses both universal and targeted prevention.

Despite an expanding body of research supporting school-based mental health interventions, several knowledge gaps remain. Although SEL and CBT-based interventions have been shown to promote mental health and alleviate subclinical symptoms of depression and anxiety among youth, the overall effectiveness of these programmes remains modest, especially in preventive programmes.^6^,^8^ Furthermore, a key limitation is the scant evidence regarding factors necessary to ensure the long-term scalability and effectiveness of such interventions.^8 16^ Importantly, at the population level, the mental health of adolescents continues to deteriorate,^17^ raising concerns about the adequacy of current approaches. Although CBT-based interventions show promise, their scalability is hindered by resource constraints, particularly the high costs of delivering targeted interventions to adolescents with sub-clinical symptoms of mental health problems.^15^ Additionally, universal programmes led by teachers may not adequately address the needs of students with emerging mental health difficulties.^13^

Given the decline in adolescent mental health and the limited effectiveness of current school-based interventions, the American Psychological Association has encouraged a change in focus from an ‘individual-level’ to a ‘population-level’ approach to mental health.^18^ Individual-level policies (i-policies) should be complemented with system-level policies (s-policies) such as community-level interventions, public policies, and system transformation to successfully improve population mental health.^19^ According to Dodge *et al*,^18^ a population-level approach would ensure mental health for diverse population groups and diminish inequalities. To successfully reach this goal, programmes should integrate three key aspects. First, programmes should scale up individual-level interventions that have been proven to be effective using implementation science approaches^18^ and make use of innovative delivery methodologies, such as the internet, mobile applications, machine learning and artificial intelligence, among others.^1820^,^22^ Second, programmes must incorporate novel methodologies and test policy-driven community-level programmes, taking successful examples from public policies promoting physical activity or abolishing corporal punishment in schools.^18^ Third, a new universal primary care model for mental health is needed.^18^

To address the aforementioned limitations of current methods and contribute to substantial advancements in the field, we are conducting the IMPROVA project. IMPROVA stands for ‘e-Intervention Enhancing Mental Health in Adolescents’. IMPROVA represents a leap forward in the field of adolescent mental health and well-being for a number of reasons.

First, the intervention programme is grounded in the principles of SEL interventions and integrates cognitive-behavioural strategies into its content. As such, IMPROVA draws on well-established, evidence-based approaches,^6^,^12^ thereby enhancing its potential effectiveness. Second, rather than addressing only mental problems, the IMPROVA programme focuses on skills and competence training to reduce barriers to access and avoid potential stigmatisation associated with using the platform.^23^ Third, the programme offers a universal intervention with some elements of a targeted intervention that is both accessible and potentially effective for adolescents with subclinical symptoms of depression and anxiety. This combination bridges the gap between universal programmes, which often lack sufficient impact for at-risk youth, and targeted interventions that require external professionals and are resource-intensive.

Fourth, IMPROVA is delivered via a scalable digital platform that enables the consistent delivery of intervention content throughout the year. This is an improvement over relying on a limited number of in-person encounters or workshops. eHealth solutions, such as mobile apps and internet platforms, have the potential to facilitate the implementation of interventions in school settings by allowing for the accessible and continuous delivery of intervention content throughout the school year.^24^ Furthermore, the persuasive design of the IMPROVA platform may increase engagement and intervention adherence, which are two of the main challenges in youth e-mental healthcare.^25^ The platform contains features such as primary task, dialogue, system credibility and social support that aim to increase engagement and adherence through personalised content, UX design, reward and reminder strategies. These features facilitate knowledge and skills acquisition and integrate social learning and social support.^26^ Moreover, compared with interventions delivered in person, eHealth platforms are more scalable and may eventually reduce the costs thanks to their potential for large-scale implementation.^27 28^ In addition, eHealth solutions, especially in school settings, may reduce the gap in access to health and educational resources caused by social inequalities^29^ since internet access is becoming more widespread, especially in the European Union (EU).^30^ Nonetheless, a systematic evaluation of commercially available mobile mental health apps for children and adolescents indicates poor overall app quality and an absence of science-driven development and evidence.^31^

Fifth, the IMPROVA programme offers tailored content for key stakeholders involved in adolescent mental health promotion, including adolescents themselves, school staff, and families. By providing user-specific resources, IMPROVA ensures that each group receives relevant guidance and tools to support mental well-being. In particular, the programme equips school staff with extensive materials to facilitate the implementation of the intervention, balancing professional support with practical application. By delivering tailored material to a diverse range of stakeholders without the need for specialised professionals and resources, IMPROVA was conceived to enhance accessibility and cost-effectiveness while preserving the quality and integrity of the intervention.

Sixth, IMPROVA engaged relevant stakeholders, including adolescents, parents, school staff and policymakers from the very beginning of the project. To enhance the programme’s cultural relevance, we conducted a series of co-creation activities—including meetings, focus groups and pilot testing—with these stakeholder groups in France, Germany, Romania and Spain. Many previous programmes implicitly assumed that vulnerable groups of adolescents (eg, at higher risk of mental health problems, from lower socioeconomic backgrounds or exposed to potentially traumatic events) would benefit most from intervention efforts. However, preliminary evidence suggests that interventions may be ineffective or even harmful in vulnerable groups, thereby increasing their risk of falling (further) behind.^23 32^ IMPROVA’s collaborative process helped tailor the intervention to varied sociocultural contexts, ensuring its acceptability, feasibility, and potential for large-scale implementation.

Taken together, the IMPROVA intervention programme was designed to enable scalable implementation across diverse educational and cultural contexts. It integrates a comprehensive mental health promotion programme within educational settings and extends its reach to adolescents’ families. By leveraging the inherent scalability of eHealth platforms, IMPROVA may reduce the logistical and financial barriers typically associated with large-scale implementation, especially for in-person programmes.^27 28^ A key feature of the programme is its modular structure, which allows for the flexible use of individual components or the full suite of resources. This enables the programme to adapt to various educational and cultural settings, user characteristics and even fluctuations in daily mood. The extensive co-creation methodology applied ensures that the programme is relevant and meaningful to a wide range of population groups, including those from lower socioeconomic backgrounds and those who are culturally underrepresented in mental health research. Through a rigorous, multi-site randomised controlled trial (RCT) and mixed-methods implementation study, IMPROVA will assess intervention effectiveness and generate critical insights into how digital, school-based mental health programmes can be effectively and equitably scaled across different populations and educational contexts.

### Objective

The primary aim of the IMPROVA RCT is to evaluate the effectiveness of the IMPROVA intervention programme in improving adolescents’ overall mental health (primary outcome). Secondary outcomes include health-related quality of life (HRQoL), depression, anxiety, social isolation, and self-esteem. This study will also examine a range of potential mechanisms (eg, adolescents’ mindsets towards change, school environment, bullying) underlying the intervention’s effects on both primary and secondary outcomes. In addition, a comprehensive assessment of the programme will be conducted, including analyses of its implementation, economic impact, and social return on investment (SROI).

## Methods and analysis

### Study design

The trial is registered with the ClinicalTrials.gov registry (NCT06556576) and the design, conduct and reporting will adhere to the Consolidated Standards of Reporting Trials^33^ and Template for Intervention Description and Replication^34^ checklists. IMPROVA is a two-arm parallel group pragmatic cluster RCT with an intervention and a wait-list control group.

### Sample size

Adolescents are the main unit of analysis of this RCT. Statistical power calculations indicated that 6000 adolescents are necessary to detect small differences (d=0.30) in mental health scores between the control and intervention groups, with 80% power. This calculation considered that schools would have 200 students (cluster size), two measurements, an aggregated intra-class correlation of schools and countries of 0.20, α=0.05, and 50% refusal to participate and loss to follow-up. A total of 6000 adolescents will also allow for stratifying the effectiveness analysis per one characteristic (eg, sex (male, female), socioeconomic status (higher, medium, lower)).

### School recruitment and selection

Following the estimations for the sample size, we aimed to recruit a total of 64 schools. The IMPROVA team invited secondary education schools in four countries: France (Ile-de-France region); Germany (Baden-Württemberg); Romania (Cluj-Napoca) and Spain (Catalonia). The strategy to select the region of the schools considered the geographical distance from the IMPROVA local team and the inputs of the regional educational administration, while ensuring the socioeconomic representativeness of the region.

The approach to schools was adapted by country, but the procedures generally followed a similar structure, summarised in the following. Schools were contacted via email and/or phone call. After a preliminary conversation (via email or phone) with school directors or a representative, the local IMPROVA team visited the schools to present the project to school directors or the board. Often, the directory board discussed the possibility of collaborating with the project with teachers and sometimes with family and student representatives before signing the collaboration contract.

Significant challenges arose at all trial sites, making it difficult to reach the target number of 64 schools. In total, 51 schools were recruited across the four sites. The breakdown by country is as follows: France: seven schools; Germany: 15 schools; Romania: 15 schools, and Spain: 13 schools. We decided to stop school recruitment since more than 20 000 students attended the 51 participating schools and were potential participants for the IMPROVA RCT. This allowed each trial site to initiate baseline assessments and implementation of the intervention. See an overview of the main challenges per trial site.

*Spain*: A prior agreement between the regional educational department and another mental well-being initiative prevented the authorities from supporting the identification and invitation of schools to participate in the IMPROVA RCT. Consequently, many of the schools in Catalonia were already part of the other mental well-being programme, preventing us from engaging with more schools.

*France*: Despite support from local educational authorities, researchers were unable to directly engage with school directors until collaboration contracts were signed by multiple stakeholders at various administrative levels. This caused further delays in school enrolment. Moreover, the questionnaire administration schedule had to take into account a tight academic schedule, which includes 2 weeks of break every 6 weeks of school.

*Romania*: The Ethical Committee did not provide feedback on the RCT protocol within the estimated timeframe because the committee was changing its board members. This delay affected the ability of the Romanian team to effectively invite and enrol participants within the timeline initially planned. Nevertheless, the Romanian team was able to begin enrolling schools and participants alongside the other trial sites.

*Germany*: Approval from regulatory bodies was delayed because the German team was required to obtain approval covering the entire federal state of Baden-Württemberg. In addition, direct access to school directors proved challenging, and schools were therefore approached via school social workers. Following initial contact, social workers were required to obtain approval from school leadership through multi-step administrative procedures, in some cases also involving parent representatives. These procedural and administrative barriers substantially delayed school recruitment.

### Blinding, randomisation and allocation concealment

A statistician external to the IMPROVA project conducted the randomisation of schools into the intervention and control groups using a computer-based random number generator. The randomisation occurred before baseline assessments since we needed to be able to tailor the explanation and information of the project to participants according to their group allocation. For example, participants in the intervention group received a more detailed explanation on how to navigate the IMPROVA platform.

As planned, the statistician conducted a block randomisation at the country level in groups of four to eight schools, depending on the number of schools recruited at each trial site (country). In France, the second round of block randomisation needed to be adapted. In France, there are two types of secondary schools with distinct age groups of adolescents (collège: 11–14 years old; lycée: 15–18 years old); therefore, in the second round of randomisation in France, the randomisation was stratified depending on the type of secondary school to ensure an equal representation of younger and older adolescents in each group.

After the randomisation, the research team and participants were not blinded regarding school allocation.

### Participants

All students enrolled in selected secondary schools were invited to participate in the project. Given the differences in educational systems across the participating countries, the age range of students varies accordingly: France (collèges and lycées, 11–18 years), Germany (gymnasium, secondary levels I and II, 10–19 years), Romania (gymnasium and lyceum, 11–18 years) and Spain (educación secundaria obligatoria, 12–16 years).

No exclusion criteria were applied. Similarly, all teachers were also invited to participate as well as the parents/legal guardians of the adolescents.

Once the schools were enrolled, participant recruitment proceeded with variations regarding rates of recruitment across countries due to national ethical regulations, particularly regarding the legal age for providing consent among adolescents.

Originally, participant recruitment was expected to be finalised in October 2024. Due to challenges in school recruitment mentioned above, the revised plan was intended to finalise participant enrolment before Christmas 2024. However, because schools had many activities (examinations, trips, seminars, etc) that prevented the completion of the baseline assessment phase before the Christmas holidays, baseline questionnaires were completed from mid-November and continued until 16 March 2025. These adaptations to the original timeline were necessary because the local project teams always tried to align their activities with the school calendar and lighten the workload and stress for the schools as much as possible.

### IMPROVA intervention

The IMPROVA intervention was developed by an interdisciplinary team of researchers and practitioners from clinical psychology, medicine, education, sociology, public health, data science and information, and communication technology, including (medical) informatics. All the content is presented in the languages of all trial sites: French, German, Romanian, Spanish, and Catalan.

Intervention development followed an iterative, multi-method approach combining top-down (theory- and evidence-based) and bottom-up (co-creation-based) strategies. The top-down process integrated SEL interventions, self-determination theory (SDT) and CBT strategies as theoretical frameworks and pre-existing intervention materials to design the first version of the programme. The bottom-up process consisted of three co-creation procedures aimed at validating and revising the initial draft of the intervention plan: (a) an expert co-creation survey with the IMPROVA consortium; (b) qualitative focus group studies with adolescents, parents, and school staff; and (c) feedback loops via local user groups. In addition, meetings with policymakers also provided feedback for the preparation of the programme. Future papers will describe the intervention development and co-creation procedures. This subsection of the current manuscript will focus on the description of the intervention programme.

### Design rationale and theoretical framework

IMPROVA is a multi-level intervention that provides tailored content for adolescents, families, and school staff. This content creates a unified and supportive framework that promotes mental health and social-emotional development among adolescents. Based on the CASEL framework,^35^ the programme addresses core SEL competencies—including self-awareness, self-management, social awareness, relationship skills and responsible decision-making—not only among students but also across the school and family environments. By actively involving important stakeholders, IMPROVA reinforces SEL principles in various settings, fostering an environment that constantly supports adolescents’ well-being and social competence holistically and sustainably.

IMPROVA takes a preventive, health-promoting approach. Disorder-specific content was intentionally excluded to avoid the need for clinical supervision, reduce stigma, enhance user acceptance, and enable scalability. In line with SDT, the programme was designed to foster intrinsic motivation and engagement by supporting adolescents’ basic psychological needs for autonomy, competence, and relatedness. The content addresses topics directly relevant to adolescents’ everyday experiences (eg, examination stress and social relationships). It draws on Cuijpers’^23^ framework of indirect interventions to increase engagement and relevance. A broad range of topics and delivery formats allows adolescents and other stakeholders to select content based on their needs and interests, thereby promoting autonomy.

In addition to its foundation in motivational and educational theory, the intervention content draws on well-established CBT strategies. These strategies include emotion regulation techniques, behavioural activation, cognitive restructuring, relaxation methods and assertiveness training, among others. These evidence-based approaches provide a practical and structured framework for developing essential emotional and social skills, complementing the programme’s broader SEL goals. The CBT-based components are adapted to be developmentally appropriate and relevant to adolescents’ everyday experiences, ensuring their accessibility and real-life applicability.

Finally, to support the above considerations and promote sustained engagement, the intervention design incorporates principles of persuasive system design (PSD),^36^ which have been demonstrated to be effective in previous digital interventions.^37 38^ Key design elements include dialogue support (such as motivational feedback, a supportive avatar and encouraging language), self-monitoring features (such as self-assessments and visual feedback on skill development), tailoring (such as content tailored to user needs and preferences) and social role modelling (such as peer narratives and case examples). The modular structure and multiple entry points (ie, users can log in and engage with the platform at their own pace, without a fixed sequence) further enhance autonomy and personalisation. The flexibility of the content and format is also intended to support implementation in diverse and often resource-constrained school settings.

### IMPROVA platform

The IMPROVA platform incorporates two key components that constitute the primary modes of delivery for the IMPROVA intervention content:

1. Information platform: an open-access psychoeducational website, available in three tailored versions for adolescents, school staff, and parents. It serves as the central access point to the programme, providing general information, guidance on available resources, and access to the interactive modules on the intervention platform.
2. Intervention platform: a password-protected eHealth platform (eSano), offering interactive training modules for adolescents and school staff.

### IMPROVA information platform

The IMPROVA information platform (https://www.improva-info.eu/en/) complements the interactive training modules available on the intervention platform and serves as an openly accessible entry point to the IMPROVA programme. It requires no login and is designed to provide quick, easy-to-navigate psychoeducational tailored content for all stakeholder groups (adolescents, families, and school staff). Although less interactive than the modules in the intervention platform, the information platform was developed to be engaging and user-friendly. Features include info boxes, dropdown menus, images, and visual figures.

Each stakeholder-specific version also contains general information about the IMPROVA project, as well as topic-specific guidance, practical tips, and links to external resources. To support users in acute distress, a prominently placed emergency help button links to a dedicated help page listing country-specific support services, including hotlines, chats, and emergency contacts. Additional help resources are embedded into topic-specific pages (eg, support for individuals affected by (cyber) bullying). Finally, the information platform functions as a bridge to the intervention platform. A dedicated sub-page introduces the interactive modules, and topic-specific pages include links to corresponding modules for adolescents and school staff who wish to explore a topic further. Table 1 presents the contents presented to adolescents, school staff, and families in the information platform.

**Table 1:** Contents of the IMPROVA information platform divided by target group

	Content for students	Content for school staff	Content for parents
1	Self-esteem	About IMPROVA	About IMPROVA
2	Strengths	Supporting students	Supporting my child
3	Emotions	Mental health (awareness)	Mental health (awareness)
4	Identity	Family-school collaborations	Family-school collaborations
5	Mental health	Tips for schools	Tips for families
6	Stress	Anti-bullying	Anti-bullying
7	Self-confidence	Child maltreatment	Child maltreatment
8	Mastering conflicts	Self-care for teachers	Self-care for parents
9	Heartache		
10	Relationships and sexuality		
11	Making friends		
12	Alcohol and drugs		
13	Sleep		
14	Physical activity		
15	Mind and body		
16	Social media		
17	Bullying		
18	School pressure and anxiety		

The adolescent version of the IMPROVA platform was developed based on the content of the modules in the intervention platform, with selected elements adapted into brief, standalone psychoeducational texts, supplemented with relevant external resources. The goal was to provide adolescents with a quick and easy way to inform themselves about key topics without requiring a login. The section for adolescents in the information platform included the tunnelling function, which is a feature that suggests content in the information platform and modules in the intervention platform based on the adolescents’ answers to a short list of questions.

For school staff and parents, the contents were based on a common set of topics defined by consortium experts and then tailored separately to the needs of each group. While the school staff version focused on school-related responsibilities and supporting students’ mental health, the parent version emphasise home-based strategies and family dynamics.

### IMPROVA intervention platform

The IMPROVA intervention platform is implemented in the eSano eHealth platform, a modular, secure, and scalable system developed for the implementation of internet- and mobile-based interventions.^39 40^ eSano enables the creation, customisation and delivery of interactive training modules through a web-based content management system and a mobile- and browser-accessible app for participants. It offers features such as multimedia content, interactive exercises, and self-monitoring tools. Designed in compliance with data protection and medical device regulations, the platform provides a flexible infrastructure for digital mental health research and practice.

The content of the IMPROVA intervention platform consists of topic-specific modules, each addressing a distinct aspect of adolescent mental health. Modules include text-based psychoeducation, interactive elements such as quizzes, case examples, and self-assessments, as well as optional ‘homework’ tasks. To further enhance engagement and support comprehension, these components are complemented by professionally produced multimedia content (eg, audio, videos, and images).

#### Modules in the intervention platform for adolescents

Table 2 presents the title and a short description of each of the 22 modules proposed to students in the intervention platform. The order presented in table 2 is also the order presented to students.

**Table 2:** Title and description of the modules for adolescents in the IMPROVA intervention platform

Module title	Module goal
Introduction I: about the eSano platform	Understand the intervention platform and its core features
Introduction II: diary function and EMA/sensing study	Learn about the EMA diary study and how to participate
Mental health: how to get your head around it	Understand mental health, common issues and how to find reliable information. Recognise early warning signs and know where to seek help for yourself and others
How to strengthen your self-esteem	Learn how self-esteem develops and explore ways to boost it. Practice strategies to handle negative self-talk
Stressed out—what now?	Reflect on your stress level and distinguish between acute and chronic stress. Learn practical coping strategies for different stressors
Keep cool under school pressure	Identify personal sources of school-related stress. Practice relaxation techniques, study strategies, and ways to manage acute performance anxiety
Exploring your emotional world	Explore your emotions and learn how to ‘ride the emotional wave’. Discover habits that support emotional well-being (eg, sleep, nutrition, exercise)
Caught in a feeling	Learn to identify and understand various emotions. Practice healthy emotion regulation strategies
Finding your inner strengths	Focus on your personal strengths and resources. Build a toolkit with practical exercises like breathing and relaxation techniques
Showing confidence in social situations	Understand how voice, tone, and posture influence confidence. Reflect on and practice these elements
Mastering conflicts and difficult conversations	Learn to recognise different types of conflict. Follow a step-by-step guide to resolve conflicts respectfully
Identity: getting to know yourself	Explore various aspects of identity. Use tools to reflect on your personality and values
Bullying: tips and support	Understand what bullying is and its effects. Learn how to support yourself and others, and debunk common myths
Test anxiety: tips and support	Identify what triggers test anxiety and how it can spiral. Learn strategies to manage it effectively
How does social media affect you?	Explore both positive and negative effects of social media. Learn strategies for balanced use, such as limiting screen time and managing Fear of Missing Out (FOMO)
Meeting new people and making friends	Gain confidence in starting conversations and making friends. Get practical tips for dating and building healthy relationships
Give your mind a break—and get moving!	Discover the link between physical activity, nutrition, and mental well-being. Learn how to add movement into your daily life
Sleep well!	Learn about healthy sleep habits and reflect on your own sleep patterns. Try personalised strategies to improve your sleep
Romantic relationships and sexuality: what you should know	Explore what makes a healthy romantic relationship. Learn about sexuality, preferences, and how to set boundaries
Heartache and how to deal with it	Understand the experience of heartache and how others cope. Learn helpful strategies and practical tips for getting through it
Alcohol, tobacco, cannabis: what you should know	Learn about short- and long-term effects of substances. Reflect on your own use and practice saying no
Problem? … Solved!	Apply structured problem-solving strategies to a personal issue. Learn how to brainstorm, evaluate, and test solutions

EMA, ecological momentary assessment.

As previously described, students’ modules include several features designed to promote engagement. These features include PSD, an avatar that accompanies the students throughout the modules, audio files, videos, and the presentation of encouraging feedback on responses provided during task completion. Social role modelling was also incorporated through, for example, peer narratives, and case examples.

#### Modules in the intervention platform for school staff

Table 3 describes the modules available for school staff in the IMPROVA intervention platform. In summary, the intervention contents offered for teachers can be subdivided into three sections:

**Table 3:** Title and description of the modules for school staff in the IMPROVA intervention platform

**No**	**Module title**	**Module goal**
0a	Introduction I: about the eSano platform	Introduces the eSano platform and its functions
1	Social and emotional learning: boosting students’ well-being	Learn the five SEL core competencies and practical classroom strategies to promote them. Understand the role of teacher modelling in fostering SEL
2	Effective communication strategies for teachers	Understand types and styles of communication, identify barriers, and learn how to actively listen and connect with students
3	Cultivating a growth mindset in students	Learn the difference between fixed and growth mindsets and discover practical ways to promote a growth mindset in your classroom
4	Understanding adolescent development: navigating change	Explore the physical, cognitive, and social changes in adolescence, and learn strategies to support students through this developmental stage
5	Nurturing yourself: self-care for teachers	Understand stress and burnout, and discover practical tools for stress management, self-awareness, and sustainable self-care practices
6	Mental health essentials: understanding and supporting students	Learn how to recognise mental health concerns, respond supportively, and follow steps for safeguarding and referral when needed
7	Empowering physical education lessons	Discover how to promote well-being through PE using the SAAFE framework. Learn how to plan, deliver, and evaluate active and inclusive lessons
8	Introduction to tutoring sessions for teachers	Learn how to run effective tutoring sessions and access eight ready-to-use session plans that can be tailored to your class

PE, physical education; SAAFE, Supportive, Active, Autonomous, Fair, Enjoyable; SEL, social and emotional learning.

- My own health: this section is designed to support school staff in managing their mental health and stress levels through a dedicated self-care module, which includes content on stress management, relaxation, and mindfulness.
- Supporting students: this section aims to help teachers support their students and improve their teaching practices. Six modules present pedagogical and health-related contents, which can be divided into two groups: (a) health-related content, including modules on adolescence and mental health awareness; and (b) pedagogical content, including modules on SEL, communication between teachers and students, growth mindset, and empowering physical education lessons.
- Tutoring sessions: eight structured lesson plans to support school staff in facilitating discussions on mental health and well-being with students during tutoring sessions, which are a common part of the curriculum across all trial sites. These sessions were designed to align with the topics covered in the students’ version of the platform. Each lesson plan includes detailed guidance and materials to help teachers lead engaging conversations on themes relevant to students’ interests and experiences. The sessions are intended to be delivered during regular classroom time (45–60 min).

Although IMPROVA is delivered through an online platform, the implementation and use of all school staff-focused content (across the three sections) are flexible. Teachers and other school staff may adapt how they engage with and apply the platform resources according to their local school context, available resources, and preferred teaching modalities.

#### Control group

During the school year 2024/2025, users in the control group continued with access to their usual care, hence not gaining access to the intervention delivered through the IMPROVA platform. After the intervention period (school year 2024/2025), adolescents, families and, school staff in the control group will be able to access the IMPROVA platform during the school year 2025/2026 (wait-list control group).

#### Intervention group

Adolescents, families, and school staff in the intervention arm have access to the IMPROVA platform, which includes both the intervention and information platforms, once baseline assessments are completed. In each participating country, a designated support person was available to provide technical assistance to users throughout the trial via email.

None of the intervention components of the IMPROVA programme was mandatory or required to be completed. Therefore, all users were free to choose which parts of the intervention programme they wished to explore or engage with. This flexible approach was designed to respect the diverse needs and contexts of users, and to promote autonomy, allowing each participant—whether adolescent, family member, or school staff—to engage with the content most relevant and useful to their specific circumstances and interests.

### Intervention implementation strategies

During the invitations for school enrolment, the local IMPROVA teams presented the project and its intervention content to school directors and teachers with administrative responsibilities (eg, head of education, coordinator). In Germany, at some schools, a social worker responsible for implementing health programmes at the school level carried out these meetings, and was the IMPROVA contact point. This difference between countries is due to the fact that IMPROVA was conceptualised to be adaptable to local characteristics. In Germany, the professionals responsible for secondary education in the region indicated that the best approach to implementing IMPROVA was through this professional.

After these presentations to the school directory board, teachers, adolescents, and families were introduced to the project during recruitment. In intervention schools, available intervention resources were highlighted. Presentation strategies were adapted to the country and school characteristics since it was imperative to adapt to local circumstances. For example, in Spain and France, the research team personally presented the project to students and school staff and conducted webinars with families. Alternative strategies included sending flyers and emails to participants.

During the school year, the local research teams had close contact with a designated school representative, who acted as the main contact point with the project. In some schools, the contact occurred via WhatsApp, while other schools preferred emails or phone calls. These interactions served to organise the distribution of communication material with teachers, students, and families of the IMPROVA intervention programme. Diverse strategies were implemented to increase the visibility of the programme, such as posting information about the IMPROVA intervention on the school website, distributing flyers via the school channels to each stakeholder, or directly sending emails to participants. Once a month, a blog post about topics of interest to adolescents (eg, ‘procrastination’ and ‘FOMO—Why it’s okay to miss out’) was uploaded to the information platform, which also promoted usage of the intervention platform. Throughout the school year, schools also received posters to display on classroom notification boards. All these strategies aimed to raise attention to the intervention programme and the number of resources available for each user type. In summary, each user type received at least one communication material from IMPROVA every month, either directly via email or indirectly (eg, posting on the school webpage, posters), aiming to disseminate the project.

### Outcomes and assessments

Overall mental health is the primary effectiveness outcome of the IMPROVA intervention programme. Secondary outcomes include HRQoL, depression, anxiety, social isolation, and self-esteem.

The assessments administered in IMPROVA include scales in various domains and were conducted at baseline (between November 2024 and March 2025), mid-term (during the intervention, March–May 2025), postintervention (immediately after the intervention, May–August 2025; primary endpoint) and 6 months postintervention (January-February 2026, secondary endpoint). Schools dedicated a school class for baseline and postintervention assessments, so teachers and/or IMPROVA staff could support students during assessments.

A description of the assessments conducted in the IMPROVA project is provided in the following sections.

#### Overall mental health

Overall mental health was assessed by the self-completion form of the Strengths and Difficulties Questionnaire (SDQ),^41^ a validated tool among adolescents aged 11–17. Adolescents rated their level of agreement with 25 items on a 3-point Likert scale ranging from 0 (‘not true’) to 2 (‘certainly true’). The 25 items represent five subscales, with scores ranging from 0 to 10: emotional symptoms, conduct problems, hyperactivity/inattention, peer problems, and prosocial behaviour.

The SDQ total score ranges from 0 to 40 points (sum of four subscales: Emotional Symptoms, Conduct Problems, Hyperactivity/Inattention, and Peer Relationship Problems). A higher score indicates poorer overall mental health. The Prosocial Behaviour Subscale score ranges from 0 to 10, and is interpreted separately. The SDQ score can also be categorised to facilitate interpretation. Initially, scores could be interpreted using a three-band categorisation: normal (0–15 points), borderline (16–19 points), and abnormal (20–40). Recently, a newer four-band categorisation has been used: close to average (0–14 points), slightly raised (15–17 points), high (18–19), and very high (20–40). Similar categorisation can also be conducted for each subscale of the SDQ.^42 43^ In addition, the SDQ also provides scores about internalising and externalising symptoms, each ranging from 0 to 20 points. The internalising score is the sum of the emotional and peer difficulties scales, whereas the externalising score is the sum of the conduct and hyperactivity scales. Finally, the SDQ contains an impact supplement, in which items on overall distress and impairment can be summed to generate an impact score that ranges from 0 to 10.

#### General HRQoL

The quality of life construct is assessed by three questionnaires: KIDSCREEN-10,^44^ the Cantril Ladder Scale^45^ and the Goal-Based Outcome Measure (GBO),^46^ since each assesses different domains of quality of life.

KIDSCREEN-10^47^ has 10 items and measures general HRQoL for adolescents. Each item on the KIDSCREEN-10 is rated on a 5-point Likert scale, reflecting the frequency or intensity of the feelings or behaviours described. The scores for the 10 items are summed to produce a total score between 10 and 50 points, where the higher the score, the higher the HRQoL. The total score can then be transformed into a T-score or percentile rank, based on normative data, to facilitate interpretation. In addition, KIDSCREEN-10 includes a general health question to provide an overview of the adolescent’s overall well-being.

The Cantril Ladder Scale^45^ consists of a visual representation of a ladder with steps numbered from 0 at the bottom to 10 at the top. Adolescents are asked to imagine that the ladder represents their life in two scenarios: (1) the present life evaluation and (2) future life expectations. The top of the ladder (step 10) represents the ‘best possible life’ they could imagine. The bottom of the ladder (step 0) represents the ‘worst possible life’ they could imagine.

The GBO^46^ is a method used to assess treatment progress and outcomes based on specific goals set by the adolescent. It is designed to capture changes in individuals’ functioning and well-being that are meaningful and relevant to them, rather than relying solely on standardised symptom measures. Adolescents are asked to select three goals they would like to achieve by the end of the current school year, and on a scale from 0 to 10, they choose the number that best describes how close they are to reaching their goal at that moment (0 means no progress has been made towards a goal, while a score of 10 means a goal has been reached fully).

#### Depression

The Patient Health Questionnaire-8 (PHQ-8)^48^ consists of eight items that correspond to the diagnostic criteria for major depressive disorder in the DSM-IV and DSM-5. Each item addresses a specific symptom of depression and is rated based on the frequency of the symptom over the past 2 weeks. Each of the items is rated on a 4-point scale. The total score ranges from 0 to 24. The total score is used to determine the severity of depression: 0–4: minimal or none, 5–9: mild, 10–14: moderate, 15–19: moderately severe, 20–24: severe. Adolescents with a score of 10 or higher will be considered as having a depressive disorder.^48^

#### Anxiety

The Generalised Anxiety Disorder-7 (GAD-7)^49^ consists of seven items that correspond to the diagnostic criteria for generalised anxiety disorder. Each item asks about the frequency of anxiety-related symptoms over the past 2 weeks. Each of the items is rated on a 4-point scale. The total score ranges from 0 to 21. The total score is used to determine the severity of anxiety: 0–4: minimal anxiety, 5–9: mild anxiety, 10–14: moderate anxiety, 15–21: severe anxiety. One question in the GAD-7 assesses the impact of anxiety on daily life.

#### Social isolation

The Social Isolation Questionnaire^50^ is composed of 17 items that can be categorised into three domains: feelings of loneliness, friendships, and family support. The score can assume values from 0 to 117, with lower scores indicating lower levels of social isolation.

#### Self-esteem

The Single-Item Self-Esteem Scale^51^ is a brief self-report measure designed to assess adolescents’ overall self-esteem with a single question. Adolescents rate their overall self-esteem on a scale ranging from one to seven, with higher numbers indicating higher self-esteem.

#### Adolescents’ mindsets towards change

For this domain, the 8-Item Implicit Theories of Intelligence Scale^52^ was administered. This scale includes eight items, four that reflect a fixed mindset and four that reflect a growth mindset. Each item is rated on a 6-point Likert scale, ranging from 1=strongly disagree to 6=strongly agree. Two separate scores (fixed and growth) and a total composite score will be obtained that can range from 8 to 48 points. A higher score represents a stronger fixed mindset, while a lower score represents a stronger growth mindset.

#### School environment

The School environment construct is assessed by two questionnaires: the Teachers and Classmate Support Scale^53^ and the 15-item School Engagement Scale.^54^

The Teachers and Classmate Support Scale^53^ is a measure designed to assess the perceived support that students receive from their teachers and classmates. It consists of two subscales: Teacher Support and Classmate Support. All items have a 5-point Likert scale. The ‘neutral’ category was not used in the IMPROVA survey. For each subscale, the scores for the individual items (1–5) are summed to obtain a total score (3–15). Higher scores indicate higher perceived support from teachers or classmates.

The 15-item School Engagement Scale^54^ is a self-report measure used to assess various dimensions of student engagement in the school environment. Developed to capture students’ psychological investment in learning and academic activities, the questionnaire evaluates multiple facets of engagement that contribute to positive educational outcomes. Scores are calculated by summing the responses across all items or within specific subscales, namely behavioural, emotional and cognitive. Higher scores indicate greater levels of engagement in the respective dimension.

#### Bullying

The revised Bully/Victim Questionnaire^55^ is a 4-item questionnaire to assess the extent to which adolescents engaged or were victims of bullying/cyberbullying. The items can be answered (and scored) on a 5-point Likert scale.

Table 4 describes the timepoint of each questionnaire and the respective domain of the IMPROVA assessment battery with adolescents.

**Table 4:** Timeline, questionnaires and respective domains that composed the main assessments with adolescents

Domain	Questionnaire	Time points			
		Baseline	Mid-term	Postintervention	Follow-up
Overall mental health	SDQ^41^	x	x	x	x
General health-related quality of life	KIDSCREEN^44^	x	x	x	x
	Cantril Ladder Scale^45^	x	x	x	x
	Goal-Based Outcome Measure^46^	x	x	x	x
Depression	PHQ-8^48^	x	x	x	x
Anxiety	GAD-7^49^	x	x	x	x
Social isolation	Social Isolation Questionnaire^50^	x	x	x	x
Self-esteem	Single-Item Self-Esteem Scale (SISE)^51^	x	x	x	x
Mindset towards change	8-Item Implicit Theories of Inteligence Scale^52^	x		x	x
School environment	Teachers and Classmate Support Scale^53^	x		x	x
	15-item School Engagement Scale^54^	x		x	x
Bullying and cyberbullying	Revised Bully/Victim Questionnaire^55^	x		x	x

GAD-7, Generalised Anxiety Disorder-7; PHQ-8, Patient Health Questionnaire-8; SDQ, Strengths and Difficulties Questionnaire.

### Demographic data and other variables

Adolescents were asked about their sex, age and date of birth, school type (eg, public, publicly financed, private), grade (school class), country of birth, who they live with (mother (only), father (only), both parents, mother/father and a partner), parental marital status, number of siblings, languages spoken at home, the area where they live (rural or urban), and the Health Behaviour in School-aged Children (HBSC) family affluence scale.^56^

#### Substance use

At baseline and postintervention, adolescents aged 15 years or above were asked about their smoking and drinking habits by the following questions: smoking in the last 30 days (number of days), average number per day of cigars/cigarettes/electronic cigarettes and similar in the last 30 days (number of units/day), drinking alcohol in the last 30 days (number of days), and drinking alcohol at least five standard doses of alcohol in the last 30 days (number of days).

#### Chronic illnesses history

Adolescents were asked if they had been diagnosed with any of the following chronic illnesses by their physician: depression, anxiety, dyslexia/attention deficit hyperactivity disorder (ADHD)/learning problems, diabetes, respiratory problems (eg, asthma or others), migraine, no, other. When the ‘other’ option was chosen, adolescents could specify the chronic illness in a free-text field.

### Ecological momentary assessment

Ecological momentary assessment (EMA) and sensor data were embedded in the app version of the eSano component of the IMPROVA platform. In January 2025, EMA asked adolescents using the app about their sleep duration and quality, current emotions, and news exposure. In addition, on submission of each EMA questionnaire, sensor data on distance travelled and step count were recorded (see the following paragraph). When students logged into the app for the first time, they were asked for permission to send notifications for the EMA questionnaires and to use the specified sensors (ie, GPS and pedometer). For each permission, an explanation was displayed as to why this permission was needed and how the data would be used. These permissions were generally optional but required for the EMA and sensing features of the app to work properly. Students were free to decide whether to reject individual or all permissions without this having any negative consequences for the app’s remaining features or the main study. If the permissions were granted, during a 2-week period, the app notified students to complete EMA questionnaires at different time points and collect mobile sensor data. In IMPROVA, a notification was sent each morning, afternoon and evening. In the morning, a slightly different questionnaire with additional questions (eg, on sleep quality) was used.

### Data from sensor measurements

Sensor data on distance travelled (GPS) and step count (pedometer) were retrieved once at each questionnaire submission and transmitted together with the EMA answers. These sensor data can be used in analyses as a proxy for physical activity and, to a certain extent, as a proxy for adherence. No continuous or background sensor data collection was performed. Instead, discrete point-in-time measurements (step count and GPS position) were transmitted exclusively at the moment of questionnaire submission. Consequently, temporal trends in physical activity or mobility can only be inferred retrospectively by computing differences across multiple submitted measurements, provided that sufficient data points are available. The GPS geolocation data collected via the eSano app is anonymised (ie, rotated around the globe) and transmitted to the eSano platform exclusively in anonymised form. Anonymised locations within an app installation are all rotated by the same, initially randomly generated amount so that the distance between them is maintained. This means that the geolocation data collected can be used to calculate the distance travelled, but does not allow any conclusions to be drawn about specific places visited, in order to protect the privacy of users. For the pedometer data, the total number of steps since the permissions were granted is continuously recorded via the mobile operating systems’ application programming interfaces and at EMA questionnaire submission, the step count is retrieved and transmitted to the eSano platform. No additional processing is performed on the sensor data, and it is stored as-is alongside the EMA answers for later data analyses. Students are able to review their submitted EMA answers in the app, but the mobile sensor data are not displayed to the students to avoid unintentional focus on these data.

### Technical data on intervention uptake (students and teachers)

Another outcome of the study is the uptake of the intervention. For the intervention platform, this includes basic usage data on how often and how participants have used the platform (eg, number of modules completed, which modules were completed). For the information website, this usage data comprises the number and sequence of user visits and the point of access (ie, the region). This information is essential to evaluate the interactions between usage and effectiveness and to assess the effectiveness of the various modules.

We also included a battery of assessments with parents, school staff, and at the school level that might provide meaningful information about the trial and could support the evaluation of the trial, such as potential mediators or moderators of effect.

### Assessments exclusive with parents

Parents were also asked to answer a number of questions: degree of kinship, family income, age (date of birth), number of children, educational level of the parents, cost data (data regarding out-of-pocket expenses related to mental health and well-being of adolescents), and their relationship with their offspring assessed by the Child-Parent Relationship Scale^57^ (CPRS-SF), which includes a subset of items rated on a Likert scale. The CPRS-SF yields scores across two key dimensions: closeness and conflict. Higher scores on the conflict subscale suggest greater relational difficulties, while higher scores on the closeness subscale indicate a more positive parent–child relationship.

The abovementioned assessments with parents occurred at baseline, except for the CPRS-SF, that will also be administered at the postintervention period. The cost data will be assessed at postintervention.

### Assessments exclusively aimed at teachers and other school staff

School staff were asked sociodemographic questions such as sex, age, years of professional experience in their current position (in years and months), level of education (highest degree), school type (public, private, etc), school size (number of students), current position (eg, headmaster, teacher, psychologist, etc), and current discipline being taught (only for teachers).

In addition, school staff also answered questions about their health (burnout, self-efficacy and stress) and their perception of the school environment (school climate). Burnout was assessed using the Maslach Burnout Inventory–Educators Survey,^58^ which measures emotional exhaustion, depersonalisation, and personal accomplishment. Self-efficacy was measured with the Teacher Sense of Efficacy Scale,^59^ evaluating teachers’ beliefs in their ability to manage classrooms, engage students, and implement instructional strategies. Perceived stress was captured using the Perceived Stress Scale,^60^ which assesses the degree to which situations in one’s life are appraised as stressful. Finally, school climate was assessed with the CLIMATEB^61^ questionnaire, which evaluates teachers’ perceptions of the school’s social and organisational environment.

Implementation determinants will be examined. These are factors or conditions that influence whether and how well an intervention or programme is implemented in a specific setting. These determinants can act as barriers or facilitators to successful implementation. Key determinants are the programme’s perceived acceptability, appropriateness, and feasibility. School staff answered the Acceptability of Intervention Measure (AIM), the Intervention Appropriateness Measure (IAM), and the Feasibility of Intervention Measure (FIM) questionnaires^62^, and questions about barriers and facilitators^63^ for implementing the IMPROVA programme. Determinants were also assessed in terms of organisational readiness for change, using a 12-item questionnaire adapted for the school setting^64^ and measured among school directors at baseline. The questionnaire assesses aspects of readiness such as willingness, commitment and, preparedness to implement IMPROVA among staff members.

The abovementioned assessments with school staff occurred at baseline, except for burnout, self-efficacy, stress, school climate, appropriateness, acceptability and feasibility, and barriers and facilitators for implementing the IMPROVA programme, which also took place during the postintervention period.

### School-wide assessments

IMPROVA also includes assessments at the school level, which will complement individual data collection, to collect information about the possible implementation of concurrent mental health/well-being programmes, cases of bullying in the last 5 years, student absenteeism, school failure, students’ grades, among other parameters. The exact list of school-wide parameters to be recorded is still being defined.

### Cost data

Parents and school directors were asked about expenses related to the mental health and well-being of adolescents (eg, costs of programmes focused on promoting mental health and well-being, costs of training, workshops or materials used in school settings, and other related family- or school-level expenses). In addition to primary data collection, cost information will be supplemented with reports from specific countries and European cost reports to ensure a comprehensive basis for economic evaluation.

### Qualitative interviews

Interviews (1×1) and focus groups (up to 10 participants) will be conducted with volunteers from all stakeholder groups (adolescents, school staff, and families after the postintervention questionnaire). These interviews/focus groups will primarily focus on inquiring with users in terms of costs, impact, usability, acceptability, appropriateness, feasibility, costs, barriers and facilitators for implementation. These interviews/focus groups will be conducted in one session and will last a maximum of 60 min with a sub-group (at least 10–15 per user type and country) of participants. The procedures will be similar regardless of the type of user or the country where the interview/focus group is taking place.

### Statistical analyses

Descriptive analyses will be performed to summarise the sociodemographic variables and scales. Frequencies and percentages will be used for categorical variables, and means, SD, medians and IQRs, minimum and maximum for continuous variables. To evaluate baseline differences between the intervention and control groups the χ^2^ test or t-test will be applied.

### Effectiveness analysis

The effectiveness of the IMPROVA programme will be evaluated based on improvements in overall mental health. Specifically, the primary analysis of effectiveness will be reflected in a reduction in the total SDQ score. In addition, effectiveness will be assessed through improvements in the secondary outcomes, as specified in a preceding section.

A linear mixed-effects model will be used to assess the effectiveness of the intervention on continuous outcomes (SDQ total score) measured across three assessment points, and generalised estimating equations will be used for categorical outcomes. Adolescents will be nested within schools, and schools will be nested within the four countries. A complementary analysis will include all four time points to evaluate the potential intervention effects at follow-up.

The model will include fixed effects for time, group (intervention vs control), and their interaction (group×time), which captures the differential change over time attributable to the intervention. The interaction term will be the main parameter of interest for assessing intervention effectiveness. To account for the multilevel design, the model will include a random intercept for each participant to account for repeated measures, a random intercept for each school to adjust for within-school clustering, and a fixed effect for country to control for systematic differences across national contexts. Other covariates associated with the outcome may be included in the model as fixed effects, depending on the objective. All models will be adjusted for the sex and age of the student.

The effectiveness analyses will be carried out following the intention-to-treat principle. In addition, a complete case analysis will be conducted as a sensitivity analysis to assess the robustness of the findings. Differences between included and excluded participants will be examined to explore potential bias due to missing data.

### Effect modification analysis

We will also evaluate whether the intervention effect varies according to certain characteristics such as participants’ sex, age, country, socioeconomic status, school type, and mental health status at baseline. These analyses will involve testing for statistically significant interaction effects between the intervention and potential moderators within linear mixed models.

### Mediation analysis

Mediation analysis will be performed using structural equation modelling to assess whether the effect of the intervention on the primary outcome is mediated by one or more intermediate variables (mediators) such as the secondary outcomes or other variables.

All available data will be included under the assumption that data are missing at random. All statistical tests will be two-sided, and a p value ≤0.05 will be considered indicative of statistical significance. Effect estimates will be reported with corresponding 95% CIs to indicate the precision of the estimates. The analysis will be conducted with SAS (V.9.4) and/or R software.

### Economic evaluation

The economic evaluation will focus on a cost-effectiveness analysis as part of the scientific publication of the IMPROVA trial. The analysis will estimate incremental cost-effectiveness ratios using validated outcome measures, including those suitable for assessing quality of life. The KIDSCREEN-10 will serve as the primary tool to derive utility values, complemented by other mental health and well-being questionnaires (see primary and secondary outcomes).

Analysis will be performed based on available study data and, where necessary, supplemented by external sources (eg, national or regional economic reports) and expert opinion. The analysis aims to capture the economic impact of the intervention, considering both costs and health outcomes.

The evaluation will be aligned, as far as possible, with the EU HTA Regulation 2021/2282 and Directive 2011/24/EU.^65^

### Implementation evaluation

IMPROVA will carry out a comprehensive analysis of implementation determinants to understand and identify factors (barriers and facilitators) influencing the implementation of the programme. To this end, perceived acceptability, appropriateness, and feasibility of the IMPROVA platform will be analysed among students, parents, teachers, and school directors. We will use the information collected in the AIM, IAM, FIM questionnaires among teachers and in qualitative interviews with all stakeholders.

Implementation outcomes will be evaluated in terms of the uptake of the IMPROVA platform. This includes basic usage data on how often and how participants have used the platform (eg, number of modules completed, which modules were completed). For the information website, this usage data comprises the number and sequence of user visits and the point of access (ie, the region). This information is essential in order to evaluate the interactions between usage and effectiveness, and to assess the effectiveness of the various modules. Adoption was measured throughout the RCT study period. Table 5 describes the implementation outcomes used to evaluate the implementation during the IMPROVA RCT.

**Table 5:** Description of the implementation outcomes in IMPROVA

**Implementation outcome**	**Definition in implementation research** ^67^	**Operationalisation of outcomes within IMPROVA**
Acceptability	The perception among implementation stakeholders that a given treatment, service, practice or innovation is agreeable, palatable or satisfactory	The perception among students, parents, teachers and school directors that the IMPROVA platform is acceptable to use
Appropriateness	The perceived fit, relevance, or compatibility of the innovation or evidence-based practice for a given practice setting, provider or consumer; and/or perceived fit of the innovation to address a particular issue or problem	The perceived fit of the IMPROVA platform to address mental health and well-being among adolescents according to students themselves, parents, teachers and school directors
Feasibility	The extent to which a new treatment or an innovation can be successfully used or carried out within a given agency or setting	The extent to which the IMPROVA platform can be successfully used within the school setting
Adoption	The intention, initial decision or action to try or employ an innovation or evidence-based practice	The uptake of the IMPROVA platform measured as initial decision to implement the intervention (eg, number of schools and individuals implementing the IMPROVA platform) and usage data among stakeholders (eg, frequency, modules completed)

### Qualitative analysis

Qualitative data are analysed separately for each co-creation procedure and for the implementation evaluation. Expert co-creation survey data was analysed descriptively and qualitatively, focusing on shared patterns in experts’ ratings and open-text responses to identify priority topics, divergent views, and cross-cutting considerations relevant to intervention development. Co-creation focus group data from adolescents, parents, and school staff first underwent a rapid qualitative analysis during the intervention development phase, using an inductive–deductive approach structured around the implementation constructs of acceptability, appropriateness, and feasibility to support timely programme refinement.^66^,^68^ Subsequently, a more in-depth analysis will be conducted using thematic analysis,^69^ involving iterative coding, theme development and comparison across stakeholder groups and trial sites. The qualitative interviews conducted as part of the RCT evaluation will also be analysed using the thematic analysis approach.

### SROI analysis of the IMPROVA programme

We will use the SROI^70^ methodology to show in a complementary manner the impact of our project. SROI is an approach to understanding and managing the impacts of the project and is a framework for measuring and accounting for the value of the project. SROI seeks to reduce inequality and improve well-being by incorporating social, environmental, and economic costs and benefits, and it measures change in ways that are relevant to the people or organisations that experience or contribute to it. It tells the story of how change is being created by measuring social, environmental, and economic outcomes and uses monetary values to represent them. This enables a ratio of benefits to costs to be calculated. For example, a ratio of 3:1 indicates that an investment of €1 delivers €3 of social value. It is especially important in a project that takes the social perspective into account, through the essential involvement of users/relatives’ associations. This analysis will be complementary to the economic evaluation.

### Patient and public involvement

Adolescents, teachers, school staff, and families were involved in the design of the IMPROVA intervention programme. A specific paper will describe all the procedures conducted to involve participants in the co-creation of the IMPROVA programme. In summary, the focus group sessions were the main co-creation activity with users. At all trial sites, stakeholders (adolescents, teachers, and school staff: at all trial sites and families: only in France and Spain) were presented with the IMPROVA intervention programme and asked to provide feedback about the topics, contents, implementation strategies, acceptability, appropriateness, feasibility, barriers and facilitators in focus group sessions with 7–15 participants from each stakeholder group in each country. A sub-group of adolescents and teachers (users’ local group) also had access to the IMPROVA intervention platform to pilot-test the platform and provide feedback about the access, registration, content, usability, and layout.

Furthermore, the feedback from representatives from the educational and health departments in each trial site was also considered in the definition of the IMPROVA programme. The policymakers’ feedback was received during meetings with the IMPROVA research team during discussions about implementing the IMPROVA RCT in the schools in each trial site (France, Germany, Romania, and Spain). Moreover, dedicated policy roundtables with all stakeholders will be held to support the sustainability and scale-up of the intervention.

### Ethics and dissemination

The study protocol of the IMPROVA RCT was approved by the ethical committee of the coordinator centre (Spain: CEIm Fundació Sant Joan de Déu, No. PIC-61-24) and the ethical boards of all trial sites (France: Comité de Protection des Personnes Ile-de-France VIII”, No. 2024-A00201-46; Germany: Ulm University Ethics Committee, No. 186-24, approval date: 5 August 2024; Romania: The Research Ethics Subcommittee of the Babeș-Bolyai University of Cluj-Napoca, No. 14.146/23.09.2024). Any amendments to the study protocols will be publicly available at the ClinicalTrials.gov Clinical Trials Registry (NCT06556576). Data management procedures will be conducted by MVM and JS. All data will be de-identified using participant codes and will be stored electronically in a secure folder at the Sant Joan de Déu Research Institute. Quality checks of entered data will be completed by MVM. Access to the final trial dataset will comply with the conditions of the ethics committees’ approval and will be at the discretion of the lead investigator, JMH.

Except in France, school principals, teachers, parents, and students provided active, informed written consent prior to enrolment. For adolescents below the legal age of consent, parental consent was required. The age of consent varied by country: 18 years in Germany, 16 in Romania, and 14 in Spain. In these countries, adolescents above the respective age thresholds could independently consent to participate. However, if a parent explicitly objected to their child’s participation, the adolescent was not permitted to take part in the study. In France, a passive consent procedure was used because IMPROVA was considered a general public health intervention: participants were given up to 2 weeks to decline participation; if no objection was received, they were considered to have consented. Of course, adolescents were free not to participate in the baseline questionnaire, even if they had not expressed their objection in the two preceding weeks. An example of the participant consent form is included in online supplemental material.

Adolescents at risk of ADHD, depression, or anxiety receive an automated notification during the assessment with information about the condition and details of public health services in their country/region. Boys with a score higher than 7 points and girls with a score higher than 5 points in the Hyperactivity/Inattention subscale were considered at risk of ADHD. Adolescents who score ≥10 points in the PHQ-8^48^ or ≥10 in the GAD-7^49^ were considered at risk of depression or anxiety, respectively.

In addition, if an adolescent mentioned serious mental health distress (self-harm, suicidal ideation, imminent bullying, or any type of abuse) during the questionnaire assessment, the trial site research team would activate the pertinent protocol in place in the country.

We plan a series of scientific peer-reviewed manuscripts evaluating the trial following the strategy described in the analytical plan in the methods section. Moreover, we plan to share dissemination material about the trial with participating schools if they are interested.

## Supplementary material

10.1136/bmjopen-2025-108674 online supplemental file 1

## References

[1] Kessler RC, Angermeyer M, Anthony JC (2007). Lifetime prevalence and age-of-onset distributions of mental disorders in the World Health Organization’s World Mental Health Survey Initiative. World Psychiatry.

[2] Mulraney M, Coghill D, Bishop C (2021). A systematic review of the persistence of childhood mental health problems into adulthood. Neurosci Biobehav Rev.

[3] WHO (2020). WHO | comprehensive mental health action plan 2013-2020-2030.

[4] (2022). Global, regional, and national burden of 12 mental disorders in 204 countries and territories, 1990–2019: a systematic analysis for the Global Burden of Disease Study 2019. Lancet Psychiatry.

[5] McGorry PD, Mei C, Dalal N (2024). The Lancet Psychiatry Commission on youth mental health. Lancet Psychiatry.

[6] Dray J, Bowman J, Campbell E (2017). Systematic Review of Universal Resilience-Focused Interventions Targeting Child and Adolescent Mental Health in the School Setting. J Am Acad Child Adolesc Psychiatry.

[7] Caldwell DM, Davies SR, Hetrick SE (2019). School-based interventions to prevent anxiety and depression in children and young people: a systematic review and network meta-analysis. Lancet Psychiatry.

[8] Clarke A, Sorgenfrei M, Mulcahy J (2021). Adolescent mental health: a systematic review on the effectiveness of school-based interventions. Early Intervention Foundation.

[9] Rasing SPA, Creemers DHM, Janssens J (2017). Depression and anxiety prevention based on cognitive behavioral therapy for at-risk adolescents: A meta-analytic review. Front Psychol.

[10] Smedler A-C, Hjern A, Wiklund S (2015). Programs for Prevention of Externalizing Problems in Children: Limited Evidence for Effect Beyond 6 Months Post Intervention. Child Youth Care Forum.

[11] Pegg S, Hill K, Argiros A (2022). Cognitive Behavioral Therapy for Anxiety Disorders in Youth: Efficacy, Moderators, and New Advances in Predicting Outcomes. Curr Psychiatry Rep.

[12] Wergeland GJH, Riise EN, Öst LG (2021). Cognitive behavior therapy for internalizing disorders in children and adolescents in routine clinical care: A systematic review and meta-analysis. Clin Psychol Rev.

[13] Shelemy DL, Harvey DK, Waite DP (2020). Meta-analysis and systematic review of teacher-delivered mental health interventions for internalizing disorders in adolescents. *Mental Health & Prevention*.

[14] Zhang Q, Wang J, Neitzel A (2023). School-based Mental Health Interventions Targeting Depression or Anxiety: A Meta-analysis of Rigorous Randomized Controlled Trials for School-aged Children and Adolescents. J Youth Adolesc.

[15] Fazel M, Hoagwood K, Stephan S (2014). Mental health interventions in schools in high-income countries. Lancet Psychiatry.

[16] Craig P, Dieppe P, Macintyre S (2008). Developing and evaluating complex interventions: the new Medical Research Council guidance. BMJ.

[17] McGorry P, Gunasiri H, Mei C (2024). The youth mental health crisis: analysis and solutions. Front Psychiatry.

[18] Dodge KA, Prinstein MJ, Evans AC (2024). Population mental health science: Guiding principles and initial agenda. *Am Psychol*.

[19] Chater N, Loewenstein G (2023). The i-frame and the s-frame: How focusing on individual-level solutions has led behavioral public policy astray. Behav Brain Sci.

[20] Schulte-Strathaus JCC, Rauschenberg C, Baumeister H (2023). Ecological Momentary Interventions in Public Mental Health Provision.

[21] Montag C, Sindermann C, Baumeister H (2020). Digital phenotyping in psychological and medical sciences: a reflection about necessary prerequisites to reduce harm and increase benefits. Curr Opin Psychol.

[22] Ebert DD, Cuijpers P, Muñoz RF (2017). Prevention of mental health disorders using internet- and mobile-based interventions: A narrative review and recommendations for future research. Front Psychiatry.

[23] Cuijpers P (2022). Universal prevention of depression at schools: dead end or challenging crossroad?. Evid Based Mental Health.

[24] Lonsdale C, Sanders T, Parker P (2021). Effect of a Scalable School-Based Intervention on Cardiorespiratory Fitness in Children A Cluster Randomized Clinical Trial. Jama Pediatr.

[25] Baumeister H, Kraft R, Baumel A (2023). Persuasive e-Health Design for Behavior Change.

[26] Valentine L, Hinton JDX, Bajaj K (2025). A meta-analysis of persuasive design, engagement, and efficacy in 92 RCTs of mental health apps. *NPJ Digit Med*.

[27] Paganini S, Teigelkötter W, Buntrock C (2018). Economic evaluations of internet- and mobile-based interventions for the treatment and prevention of depression: A systematic review. J Affect Disord.

[28] Wu Q, Li J, Parrott S (2020). Cost-Effectiveness of Different Formats for Delivery of Cognitive Behavioral Therapy for Depression: A Systematic Review Based Economic Model. Value Health.

[29] Honeyman M, Maguire D, Evans H (2020). Digital Technology and Health Inequalities: A Scoping Review.

[30] EUROSTAT (2024). Digital economy and society statistics - households and individuals - statistics explained - eurostat. https://ec.europa.eu/eurostat/statistics-explained/index.php?title=Digital_economy_and_society_statistics_-_households_and_individuals.

[31] Domhardt M, Messner EM, Eder AS (2021). Mobile-based interventions for common mental disorders in youth: a systematic evaluation of pediatric health apps. Child Adolesc Psychiatry Ment Health.

[32] Montero-Marin J, Allwood M, Ball S (2022). School-based mindfulness training in early adolescence: what works, for whom and how in the MYRIAD trial?. Evid Based Ment Health.

[33] Moher D, Hopewell S, Schulz KF (2010). CONSORT 2010 explanation and elaboration: updated guidelines for reporting parallel group randomised trials. BMJ.

[34] Hoffmann TC, Glasziou PP, Boutron I (2014). Better reporting of interventions: template for intervention description and replication (TIDieR) checklist and guide. BMJ.

[35] CASEL (2020). CASEL’s sel framework. https://casel.org/fundamentals-of-sel/what-is-the-casel-framework/.

[36] Oinas-Kukkonen H, Harjumaa M (2009). Persuasive Systems Design: Key Issues, Process Model, and System Features. *CAIS*.

[37] Mutter A, Küchler AM, Idrees AR (2023). StudiCare procrastination - Randomized controlled non-inferiority trial of a persuasive design-optimized internet- and mobile-based intervention with digital coach targeting procrastination in college students. BMC Psychol.

[38] Küchler A-M, Schultchen D, Dretzler T (2023). A Three-Armed Randomized Controlled Trial to Evaluate the Effectiveness, Acceptance, and Negative Effects of *StudiCare Mindfulness,* an Internet- and Mobile-Based Intervention for College Students with No and “On Demand” Guidance. Int J Environ Res Public Health.

[39] Kraft R, Idrees AR, Stenzel L ESano – an ehealth platform for internet- and mobile-based interventions.

[40] Idrees AR, Kraft R, Pryss R (2022). Backend concept of the esano ehealth platform for internet- and mobile-based interventions.

[41] Goodman R, Meltzer H, Bailey V (1998). The Strengths and Difficulties Questionnaire: a pilot study on the validity of the self-report version. Eur Child Adolesc Psychiatry.

[42] Goodman A, Goodman R (2009). Strengths and difficulties questionnaire as a dimensional measure of child mental health. J Am Acad Child Adolesc Psychiatry.

[43] SDQ (2025). What is the SDQ?. https://www.sdqinfo.org/a0.html.

[44] Ravens-Sieberer U, Erhart M, Rajmil L (2010). Reliability, construct and criterion validity of the KIDSCREEN-10 score: a short measure for children and adolescents’ well-being and health-related quality of life. Qual Life Res.

[45] Levin KA, Currie C (2014). Reliability and Validity of an Adapted Version of the Cantril Ladder for Use with Adolescent Samples. Soc Indic Res.

[46] Duncan C, Cooper M, Saxon D (2023). Test-retest stability, convergent validity, and sensitivity to change for the Goal-Based Outcome tool for adolescents: Analysis of data from a randomized controlled trial. J Clin Psychol.

[47] Ravens-Sieberer U, Erhart M, Rajmil L (2010). Reliability, construct and criterion validity of the KIDSCREEN-10 score: a short measure for children and adolescents’ well-being and health-related quality of life. Qual Life Res.

[48] Kroenke K, Strine TW, Spitzer RL (2009). The PHQ-8 as a measure of current depression in the general population. J Affect Disord.

[49] Mossman SA, Luft MJ, Schroeder HK (2017). The Generalized Anxiety Disorder 7-item scale in adolescents with generalized anxiety disorder: Signal detection and validation. Ann Clin Psychiatry.

[50] Dos Santos SJ, Soares FC, Gaoua N (2024). Development and validation of a scale to measure social isolation in adolescents. J Res Adolesc.

[51] Robins RW, Hendin HM, Trzesniewski KH (2001). Measuring Global Self-Esteem: Construct Validation of a Single-Item Measure and the Rosenberg Self-Esteem Scale. *Pers Soc Psychol Bull*.

[52] Dweck CS (2013). Self-theories: Their Role in Motivation, Personality, and Development. Self-Theories.

[53] Torsheim T, Wold B, Samdal O (2000). The Teacher and Classmate Support Scale: Factor Structure, Test-retest Reliability and Validity in Samples of 13- and 15-Year-Old Adolescents. Sch Psychol Int.

[54] Fredricks JA, Blumenfeld P, Friedel J (2005). School Engagement. What Do Child Need to Flourish.

[55] Paniagua C, García-Moya I, Sánchez-Queija I (2022). Bullying, cyberbullying, and adoption: What is the role of student-teacher connectedness?. *Sch Psychol*.

[56] Torsheim T (2019). HBSC Family Affluence Scale Coding Guidance (V1): HBSC Methods.

[57] Driscoll K, Pianta RC (2021). Child-Parent Relationship Scale. PsycTESTS Dataset.

[58] Maslach C, Jackson SE, Leiter M (2015). The Maslach Burnout Inventory Manual.

[59] Tschannen-Moran M, Hoy AW (2001). Teacher efficacy: capturing an elusive construct. *Teaching and Teacher Education*.

[60] Cohen S, Kamarck T, Mermelstein R (1983). A global measure of perceived stress. J Health Soc Behav.

[61] Veletić J, Price HE, Olsen RV (2023). Teachers’ and principals’ perceptions of school climate: the role of principals’ leadership style in organizational quality. *Educ Asse Eval Acc*.

[62] Weiner BJ, Lewis CC, Stanick C (2017). Psychometric assessment of three newly developed implementation outcome measures. Implementation Sci.

[63] Robinson CH, Damschroder LJ (2023). A pragmatic context assessment tool (pCAT): using a Think Aloud method to develop an assessment of contextual barriers to change. *Implement Sci Commun*.

[64] Arthur K, Christofides N, Nelson G (2020). Educators’ perceptions of organisational readiness for implementation of a pre-adolescent transdisciplinary school health intervention for inter-generational outcomes. PLoS One.

[65] Europian Union Regulation (eu) 2021/2282 of the european parliament and of the council of 15 december 2021 on health technology assessment and amending directive 2011/24/eu. https://eur-lex.europa.eu/legal-content/EN/TXT/?uri=CELEX:32021R2282.

[66] Schultes MT (2023). An introduction to implementation evaluation of school-based interventions. European Journal of Developmental Psychology.

[67] Proctor E, Silmere H, Raghavan R (2011). Outcomes for Implementation Research: Conceptual Distinctions, Measurement Challenges, and Research Agenda. *Adm Policy Ment Health*.

[68] Vindrola-Padros C, Chisnall G, Polanco N (2022). Iterative Cycles in Qualitative Research: Introducing the RREAL Sheet as an Innovative Process. *SSRN Journal*.

[69] Braun V, Clarke V (2006). Using thematic analysis in psychology. Qual Res Psychol.

[70] Social Value International (2012). The guide to sroi. https://www.socialvalueint.org/guide-to-sroi.

