# Supplementary material for: Study protocol of the e-Intervention Enhancing Mental Health in Adolescents (IMPROVA) project: a randomised controlled trial to promote adolescents’ mental health and well-being in four European countries
Source: BMJ Open. 2026 Apr 28;16(4):e108674. doi: 10.1136/bmjopen-2025-108674 (PMC13141090; doi:10.1136/bmjopen-2025-108674)
Supplement: online supplemental file 1 [file bmjopen-16-4-s001.pdf]

Supplemental Material

Below is an example of the participant consent form for adolescents in the intervention group in Catalan. Except in France, where a passive informed consent procedure was applied, a similar version of the consent form was available in participants' preferred language (German, Romanian, or Spanish). Furthermore, adolescents in the control group, as well as school staff and families in the intervention and control groups, received a tailored version of the participant consent form.

INFORMATION SHEET

IMPROVA: e-Intervention Enhancing Mental Health in Adolescents

Et convidem a participar en l'estudi Projecte IMPROVA finançat per la Comissió Europea (nombre de projecte: 101080934).

Abans de donar el teu consentiment per participar en l'estudi, és essencial que comprenguis en què consisteix. Si us plau, llegeix atentament aquesta informació i realitza totes les preguntes que consideris necessàries.

Què és IMPROVA?

IMPROVA és una innovadora aplicació online i una pàgina web dissenyada per promoure el creixement personal i el benestar dels/les adolescents. A més, proporciona a docents, personal escolar i famílies d'habilitats i coneixements per donar suport eficaçment a l'alumnat.

Ofereix informació senzilla sobre diversos temes —des de la gestió de les emocions i l'estrès fins a les relacions interpersonals—, IMPROVA és una eina completa per promoure la salut entre els/les adolescents!

Els/les adolescents, el professorat, el personal del centre educatiu i les famílies poden accedir a IMPROVA des de qualsevol dispositiu, ja sigui un mòbil, un ordinador o una tauleta digital. El nostre objectiu és crear un espai dinàmic que vagi més enllà dels consells genèrics, amb eines pràctiques i continguts recolzats per persones expertes. IMPROVA pretén dotar els usuaris de coneixements i habilitats per millorar el benestar emocional, fomentar relacions sanes i afrontar els reptes amb solvència.

Com a plataforma senzilla, IMPROVA està dissenyada per ser interactiva i atractiva, fent que l'experiència d'aprenentatge sigui agradable per a tothom. Amb seccions dedicades als/les adolescents, al professorat i al personal escolar, i a les famílies, IMPROVA adapta el seu contingut per satisfer les necessitats i interessos propis de cada grup.

Quin és l'objectiu de l'estudi IMPROVA?

L'objectiu d'aquest estudi és saber si la plataforma IMPROVA ajuda a millorar el benestar de les persones joves, com tu. Volem veure si l'ús d'aquesta nova aplicació i pàgina web marca una diferència en la vida dels/les adolescents, les seves famílies i docents.

Per a això, demanarem a instituts de França, Alemanya, Romania i Espanya que s'uneixin al nostre estudi. A continuació, els dividirem aleatòriament en dos grups. Un grup seleccionat a l'atzar tindrà accés a la plataforma IMPROVA, la qual cosa permetrà a alumnat, professorat i famílies utilitzar els seus continguts i recursos com a part del seu sistema de suport. L'altre grup, denominat grup de control, serà també elegit a l'atzar i continuarà amb la seva rutina habitual, sense tenir accés al programa IMPROVA. Els usuaris d'ambdós grups rebran un informe sobre la seva salut i benestar. No et preocupis, només tu tindràs accés a l'informe!

Els instituts s'assignen aleatòriament a diferents grups. Amb aquest mètode, podrem mesurar i

comparar en quina mesura IMPROVA promou salut en comparació amb la rutina habitual dels centres educatius. Aquest disseny és crucial per esbrinar si IMPROVA realment marca una diferència, i és una eina útil de suport a les rutines escolars i al dia a dia de l'adolescent. Això ens ajudarà a arribar a conclusions sobre la possible eficàcia d'IMPROVA en la promoció de la salut mental i el benestar entre els/les adolescents, juntament amb les seves famílies i docents.

Què passarà si participo en l'estudi?

El teu institut ha estat assignat al grup que utilitzarà la plataforma IMPROVA. Això significa que se t'oferirà a tu, als teus docents i al personal del centre, així com a la teva família, l'oportunitat d'utilitzar la plataforma IMPROVA. Però, no et preocupis, cada grup tindrà la seva pròpia secció amb informació individualitzada. A més, el professorat, el personal del col·legi i les famílies no veuran res sobre com utilitzes IMPROVA: només tu tindràs accés a aquesta informació.

Si decideixes participar en aquest estudi, la teva participació durarà aproximadament un curs escolar. Durant aquest temps, se t'animarà a utilitzar l'aplicació IMPROVA i el lloc web tant com vulguis.

A més, se't demanarà que emplenis qüestionaris sobre els teus pensaments, sentiments i objectius relacionats amb l'ús de l'aplicació IMPROVA. Això et portarà aproximadament 30 minuts cada vegada, i et demanarem que completis aquests qüestionaris en línia en quatre moments:

1. Setembre-octubre de 2024 (abans de començar a utilitzar la plataforma IMPROVA)

2. Febrer de 2025 (a meitat del curs escolar)

3. Juny de 2025 (a la fi del curs escolar)

4. Desembre de 2025

A més del qüestionari, t'animem que comparteixis en la plataforma els teus pensaments, sentiments i qualsevol repte al qual t'enfrontis com a part de la intervenció IMPROVA. També és essencial tenir en compte que, encara que aquests exercicis són crucials per a la intervenció, la informació que proporcions en la plataforma no serà revisada per professionals de la salut mental (per exemple, psicòleg/loga, psiquiatre).

Beneficis:

Si participes, rebràs un informe sobre la teva salut i benestar. A més, et facilitarem les dades (telèfon, pàgina web i adreça) on pots buscar suport en els serveis sanitaris locals del teu entorn si identifiquem que et podries beneficiar d'un suport addicional.

També contribuiràs al desenvolupament i millora de la plataforma i la intervenció IMPROVA. Això, alhora, té el potencial de promoure eficaçment la salut mental i el benestar entre els/les adolescents, les seves famílies i docents d'arreu d'Europa. La teva participació pot tenir un impacte positiu en un nombre significatiu d'adolescents.

Riscos:

No es preveuen desavantatges associats a la participació en aquest estudi. No estàs obligat/da respondre a cap pregunta que no desitges contestar, i tens la llibertat d'interrompre la teva participació en qualsevol moment si decideixes no continuar.

Participació voluntària:

La teva participació en aquest estudi és totalment voluntària. Tens la llibertat de triar no participar o canviar la teva decisió, retirant-te en qualsevol moment sense necessitat de donar més explicacions o motius.

Què passa si hi ha algun problema?

Si trobes algun problema durant l'estudi o tens seriosos dubtes sobre la teva salut o benestar, pots posar-te en contacte amb l'equip de l'estudi en: parcsanitari.improva@sjd.es.

Si no estàs satisfet/a, també pots posar-te en contacte amb: parcsanitari.improva@sjd.es.

Si experimentes alguna incidència mèdica durant l'estudi, preguem que informis al més aviat possible a l'equip de l'estudi a parcsanitari.improva@sjd.es.

Què haig de fer si m'interessa participar?

Si decideixes participar, demanarem el teu consentiment, que pots proporcionar fent clic en l'opció de baix que consent a participar en IMPROVA.

Si tens alguna pregunta o desitges comentar la teva participació abans de prendre una decisió, no dubtis a posar-te en contacte amb el nostre equip a parcsanitari.improva@sjd.es.

Confidencialitat i protecció de dades:

La teva privacitat és important. Les teves respostes als qüestionaris seran confidencials i s'emmagatzemaran de forma anònima. Quan t'uneixis a l'estudi, es t'assignarà un número d'identificació únic, de manera que el teu nom no es relacionarà amb les dades de la investigació. Podrem compartir les dades anonimitzades amb institucions de fora de la Unió Europea per a finalitats de recerca diferents de les especificades en l'estudi IMPROVA. Cal destacar que tota la informació recopilada té estrictament finalitats d'investigació i s'emmagatzemarà seguint la Llei de Protecció de Dades de 2018 i el Reglament General de Protecció de Dades (GDPR) de la Unió Europea. Qualsevol troballa només s'informarà de "forma agregada", assegurant que no puguis ser identificat/da en cap informe o publicació. Una còpia de la informació de consentiment informat amb evidència d' acceptació i les dades recollides en l'estudi es mantindran en custòdia del Parc Sanitari Sant Joan de Déu durant un període de 10 anys.

Podràs exercir els teus drets d'Accés, Rectificació, Supressió ("dret a l'oblit"), Oposició i qualsevol altre dret reconegut en els termes i condicions establerts en la legislació vigent en matèria de Protecció de Dades (LOPD-GDD 3/2018, GDPR-UE 679/2016), com ara el dret a la

limitació del tractament, a la portabilitat de les dades, a no ser objecte de decisions individuals automatitzades, inclosa l'elaboració de perfils, així com revocar l'autorització per a la inclusió en l'estudi.

Per a l'exercici d'aquests drets, hauràs d'adreçar-te, personalment o per escrit, indicant clarament la teva sol·licitud i adjuntant còpia del Document d'Identificació (DNI/NIE/Targeta Id./Passaport equivalent) a l'adreça de correu electrònic: parcsanitari.improva@sjd.es o per correu postal: Projecte IMPROVA, Unitat de Recerca, Parc Sanitari Sant Joan de Déu (PSSJD), Dr. Antoni Pujadas 42, 08830 Sant Boi de Llobregat. La institució responsable de les dades és Parc Sanitari Sant Joan de Déu. En cas de no estar d'acord amb el tractament de les teves dades o amb l'exercici dels teus drets, en primera instància, pots dirigir-te per escrit al Delegat de Protecció de Dades del PSSJD a través de la Sra. Maria Martí: oficina_dpd@pssjd.org. En segon lloc, pots enviar una reclamació a l'Autoritat de Control (Autoritat Catalana de Protecció de Dades: https://apdcat.gencat.cat/ca/drets_i_obligacions/reclamar_i_denunciar).

Mantenim la privacitat de la teva informació i es respectarà la confidencialitat amb subjecció a les limitacions legals i les directrius professionals. Tots els membres de l'equip de recerca han rebut formació en matèria de protecció de dades i són conscients que existeixen circumstàncies particulars en les quals s'ha de trencar la confidencialitat. En cas que sorgeixin dubtes sobre un possible dany o perill per a un/a mateix/a o per a una altra persona, pot ser necessari trencar la confidencialitat, ja que s'han de seguir els procediments de salvaguarda. Si això ocorregués, l'equip investigador seguiria els seus procediments de salvaguarda.

Què passarà amb els resultats de l'estudi de recerca?

Pots mantenir-te informat/da periòdicament sobre l'evolució de la investigació a través d'un butlletí informatiu de l'estudi. Si desitges rebre el nostre butlletí, fes clic aquí.

Quan finalitzi l'estudi, tenim previst publicar els nostres resultats en revistes científiques i podrem presentar els resultats anonimitzats en conferències científiques o a qualsevol altra part interessada (associacions d'estudiants/professorat/educadors/es, responsables polítics, periodistes, etc.). Les dades recollides també es podran utilitzar com a part d'estudis de recerca de doctorat. En tot moment les teves dades seran totalment anònimes: cap publicació o informe d'investigació t'identificarà individualment.

T'enviarem un resum de l'informe d'investigació en un termini de 12 mesos a partir de la data de finalització de l'estudi. Si desitges una còpia de la investigació publicada, fes-nos-ho saber i estarem encantats/des d'enviar-te qualsevol publicació que descrigui els resultats d'aquesta investigació a mesura que estiguin disponibles.

Finançament: El projecte IMPROVA ha rebut finançament de la Comissió Europea (Projecte núm 101080934).

Preguntes / altra informació: Si desitges fer preguntes o aclarir qualsevol qüestió relacionada amb l'estudi, no dubtis a posar-te en contacte amb l'equip investigador parcsanitari.improva@sjd.es.

L'equip de recerca agraeix la teva valuosa col·laboració.

CONSENTIMENT INFORMAT

IMPROVA: e-Intervention Enhancing Mental Health in Adolescents

Si desitges participar en el projecte IMPROVA, llegeix atentament les Fitxes Informatives i la següent informació. Si tens alguna pregunta abans de participar en l'estudi, pots posar-te en contacte amb el nostre equip; correu electrònic: parcsanitari.improva@sjd.es o per correu postal: Projecte IMPROVA, Unitat de Recerca, Parc Sanitari Sant Joan de Déu (PSSJD), Dr. Antoni Pujadas 42, 08830 Sant Boi de Llobregat.

He estat convidat/da a participar en l'estudi d'investigació IMPROVA i després de rebre la corresponent informació sobre el projecte, DECLARO que:

- He llegit i comprès el Full Informatiu de l'esmentat estudi. He tingut l'oportunitat de considerar la informació i el que s'espera de mi. També he tingut l'oportunitat de formular preguntes que han estat respostes satisfactòriament.
- M'agradaria participar en l'estudi IMPROVA i entenc que la meva participació en l'estudi és totalment voluntària. Soc lliure de retirar la meva participació en qualsevol moment sense haver d'oferir més explicacions o raons per fer-ho, sense que això afecti els meus drets sanitaris o legals.
- En revisar el Full Informatiu, he estat informat/da sobre com les dades que facilito seran utilitzades i emmagatzemades pel processador de dades (Parc Sanitari Sant Joan de Déu, PSSJD). Soc conscient dels meus Drets d'Accés, Rectificació, Supressió ("dret a l'oblit"), Oposició, i qualssevol altres drets estipulats en els termes i condicions assenyalats per la legislació vigent en matèria de Protecció de Dades (GDPR-UE 679/2016). Això inclou drets com la limitació del tractament, la portabilitat de les dades, el dret a no ser objecte de decisions individuals automatitzades, inclosa l'elaboració de perfils, i el dret a revocar l'autorització per a la inclusió en l'estudi.
- Entenc que la informació que faciliti serà analitzada amb finalitats de recerca, que poden incloure —però no es limiten— al projecte IMPROVA, i dono el meu consentiment per a aquest ús.
- Atès que el projecte IMPROVA és d'àmbit internacional, reconec que les dades anonimitzades poden ser compartides fora d'Espanya amb els socis de l'estudi, i dono el meu consentiment per a aquesta utilització.

Ara que has llegit el full d'informació i el consentiment informat, pots tornar al enllaç del formulari i omplir les teves dades personals per a consentir la teva participació al projecte.
